# Spinal fusion in facioscapulohumeral dystrophy for hyperlordosis: A case report

**DOI:** 10.1097/MD.0000000000018787

**Published:** 2020-02-21

**Authors:** İlker Eren, Berk Abay, Caner Günerbüyük, Özgür Öztop Çakmak, Cüneyt Şar, Mehmet Demirhan

**Affiliations:** a Koc University, School of Medicine, Department of Orthopaedics and Traumatology; b Koc University, School of Medicine; c Koc University, School of Medicine, Department of Neurology; d Istanbul University, Istanbul Faculty of Medicine, Department of Orthopaedics and Traumatology, 34010, Istanbul, Turkey.

**Keywords:** facioscapulohumeral muscular dystrophy, hyperlordosis, muscular dystrophy, quality of life, spinal fusion

## Abstract

**Rationale:**

Facioscapulohumeral muscular dystrophy (FSHD) is the third most common muscular dystrophy, which is associated with facial, shoulder girdle, and paraspinal muscle atrophy. Most of the patients develop hypokyphosis and hyperlordosis in the course of the disease, to preserve standing posture. Corrective fusion is contraindicated in these patients as the surgery results with loss of compensatory hyperlordosis and leads to loss of trunk balance while standing. Although spinal fusion in neuromuscular scoliosis is a known treatment option, there are no studies in the literature on the spinal fusion of this specific patient group.

**Patient concerns:**

In this case report we have presented a 66-year-old woman, who was admitted with back and abdominal pain, inability to sit straight, abdominal discomfort, and numbness in the lower extremities after prolonged sitting.

**Diagnoses:**

The patient developed severe hyperlordosis causing intra-abdominal disorders, radicular symptoms, and sitting discomfort due to FSHD.

**Interventions:**

The patient underwent T2–S1 fusion and successful fusion was achieved.

**Outcomes:**

Individualized Neuromuscular Quality of Life Questionnaire (INQoL) was used to assess preoperative and 3 years postoperative functional outcomes. All domains and total score improved at the end of the follow-up period and successful fusion was verified radiologically.

**Lessons:**

This case suggests that spinal fusion may provide functional improvement in carefully selected patient groups. Patient stratification considering spinal disability is required for further studies in this specific indication.

## 1 Introduction

Facioscapulohumeral muscular dystrophy (FSHD) is the third most common muscular dystrophy with autosomal dominant inheritance and prevalence of 1/15.000 to 1/20.000.^[1–5]^ Clinical signs may show great variability, but most of the patients develop the characteristic facial, shoulder girdle, periscapular, paraspinal, and pelvic weakness.^[6,7]^ Abdominal and lower extremity muscles are also affected. In their lifetime, 20% to 37% of these patients become wheelchair bound.^[8]^

Sagittal spinal imbalance due to pelvic extensor and paraspinal muscle weakness leads to the characteristic hyperlordosis deformity. Although it is a well-known compensation in FSHD, it has not been studied in depth.^[9]^ Correcting these compensatory changes may lead to loss of trunk balance and corrective surgeries are not indicated in mobile patients.^[10]^

Spinal deformities associated with neuromuscular disorders, such as scoliosis, hyperlordosis, or pelvic obliquity are well known topics and both surgical and non-surgical treatment options are extensively discussed in the literature.^[11–14]^ Spinal fusion is considered as a successful treatment option which restores the quality of life and prevents progression. Currently, there is a single reported case in the literature which was published recently with a good result in a selected patient.^[8]^ There are no reports consisting of case series or comparative studies.

In this report, we have presented the spinal fusion of a wheelchair dependent patient with severe hyperlordosis associated with FSHD, causing abdominal discomfort and radicular symptoms. Successful fusion was achieved, and the postoperative clinical score showed improvement in the quality of life.

## 2 Case report

Before commencing the study, written consent was obtained from the patient for publication of clinical findings, preoperative, and postoperative photos.

A 66-year-old woman was admitted with back and abdominal pain, inability to sit straight, abdominal discomfort, and numbness in the lower extremities after prolonged sitting. Her first complaints, face and upper extremity weakness, started when she was 18 years old. She was diagnosed with FSHD clinically at the age of 21 when her lower extremity weakness started. Her lumbar lordosis increased gradually. She became wheelchair dependent at the age of 44. She suffered right femoral neck fracture after a fall injury at 47, which limited her mobility and daily activities further. Her hip fracture was not operated. She has had abdominal complaints and lower extremity numbness for the last 5 years. She was not diagnosed with any specific gastrointestinal (GI) disorders.

She was operated due to left breast cancer at the age of 45, used tamoxifen for 5 years after the surgery. Difficulties with breathing started at the age of 60 and she has started using bilevel positive airway pressure device (BIPAP) since the age of 63. In her family, her maternal grandmother, mother, sister, and single daughter were diagnosed with FSHD as well. She was working as a columnist for a national newspaper for the last 8 years. Her daily practice was particularly affected due to discomfort with prolonged sitting.

**Figure 1:**
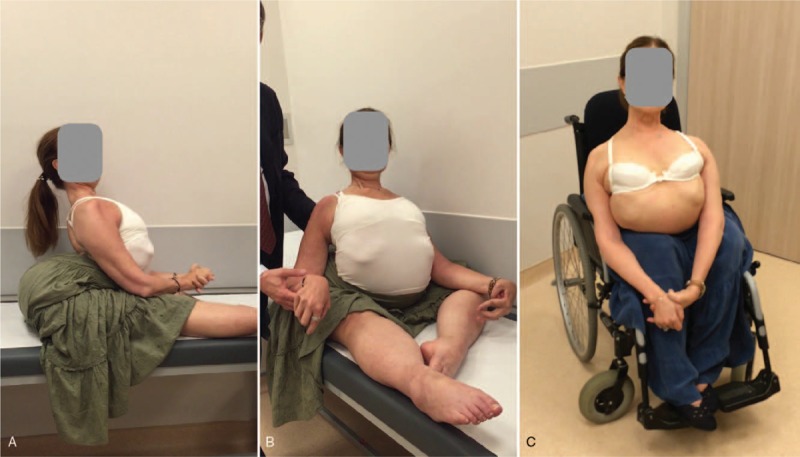
Preoperative clinical pictures of the patient. She has extreme hyperlordosis during sitting as shown from side (A) and front (B). Although there is a back support, she was unable to obtain a straight posture on wheelchair (C).

The patient was unable to walk or stand. She was able to sit without support with hypokyphosis and extreme hyperlordosis (Fig. 1). Her spinal deformities were noted as flexible with remaining mild hyperlordosis in supine position. Facial muscle atrophy was consistent with FSHD. Her muscle strength using Medical Research Council (MRC) Scale were measured as: neck extension 3/5, neck flexion 4/5, deltoid bilateral 0/5, elbow flexion bilateral 3/5, elbow extension bilateral 0/5, wrist extension bilateral 0/5, wrist flexion bilateral 3/5, hip and knee flexors and extensors bilateral 0/5, ankle dorsiflexion 0/5, ankle plantarflexion 3/5. Beevor's sign was positive. No scapular winging was observed. Four centimeters of lower extremity shortening was measured on her right side.

FSHD evaluation scale^[15]^ was calculated as: I – facial weakness 2 points, II – scapular girdle involvement 3 points, III – upper limbs involvement 2 points, IV – legs involvement 2 points, V – pelvic girdle involvement 5 points, VI – abdominal muscle involvement 1 point, with the total point of 15.

Quality of life before and after surgery was documented using Individualized Neuromuscular Quality of Life Questionnaire (INQoL) Version 2.0.^[16]^ Preoperative INQoL scores in the symptoms domain were calculated as: I – weakness 100%, II – pain 94.74 points, III – fatigue 94.74 points, IV – muscle locking 0 points, V – droopy eyelids 47.37 points, VI – double vision 0 points, VII – swallowing difficulties 63.16 points. Life domain scores were calculated as: I – activities 76.85 points, II – independence 97.22 points, III – social relationships 44.44 points, IV – emotions 19.44 points, V – body image 88.88 points. Her expected treatment score was 83.33 and her quality of life score was 55.55 (Table 1).

**Table 1:**
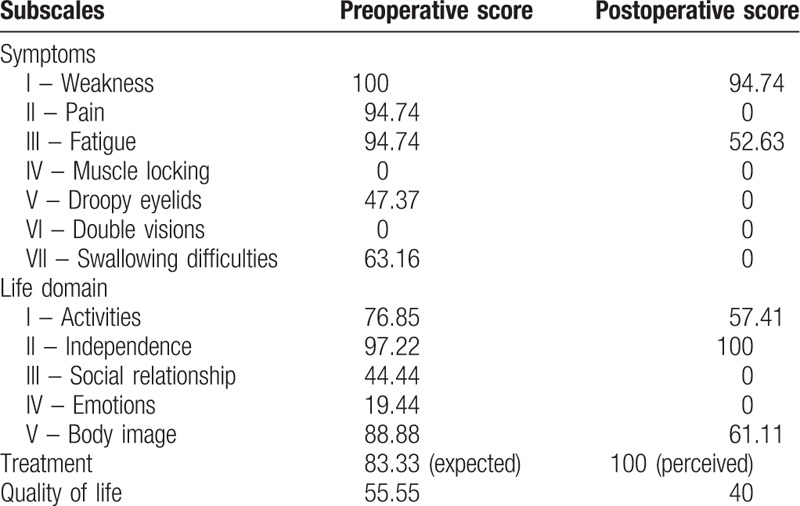
Subdomain and subscale score distribution of “Individualized Neuromuscular Quality of Life” (INQoL) score. Lower scores indicate better clinical impact for all domains and subscales, except treatment domain where higher score indicates better clinical impact.

**Figure 2:**
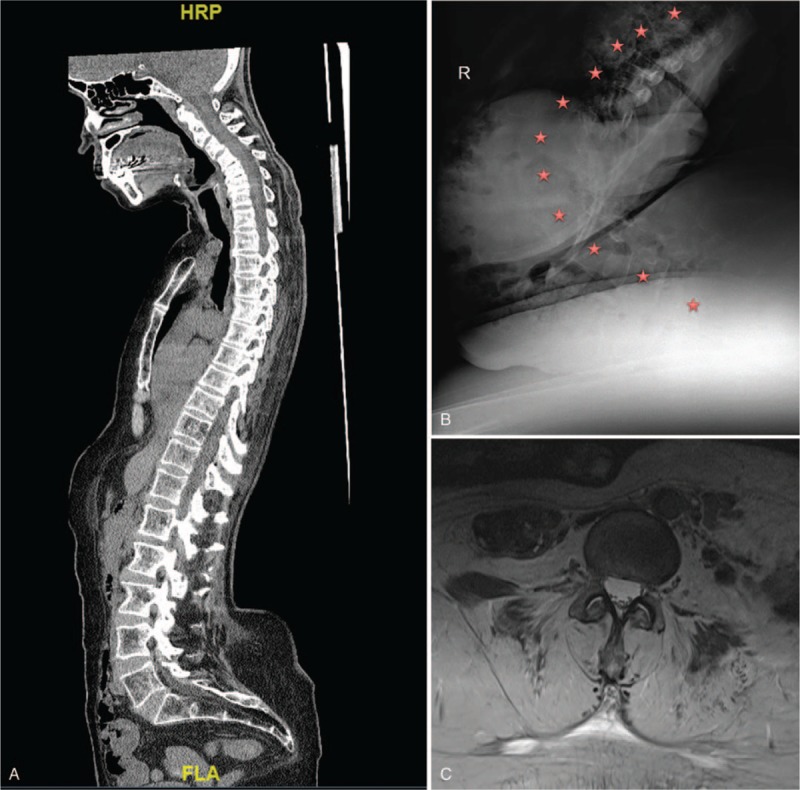
Her radiologic assessment in supine revealed that her deformities were completely flexible, as shown in the sagittal plane computerized tomography scan (A). Her deformity was obvious on lateral lumbar X-ray while sitting. Vertebrae corpuses are marked in red (B). Preoperative MRI revealed severe paraspinal muscle atrophy (C).

Lateral sitting, supine antero-posterior (AP) and lateral X-rays, computerized tomography (CT), and magnetic resonance imaging (MRI) of the whole spine were obtained. Lateral spinal X-rays in sitting position revealed hypokyphosis and extreme hyperlordosis which was flexible as shown in Figure 2A and B. Paraspinal muscle atrophy was observed in MRI (Fig. 2C). No myelopathy or radiculopathy was noted on any level. Electromyography (EMG) showed non-specific myopathic changes in both upper and lower extremities and no findings associated with radiculopathy. EMG during sitting was not performed.

### 2.1 Surgery

**Figure 3:**
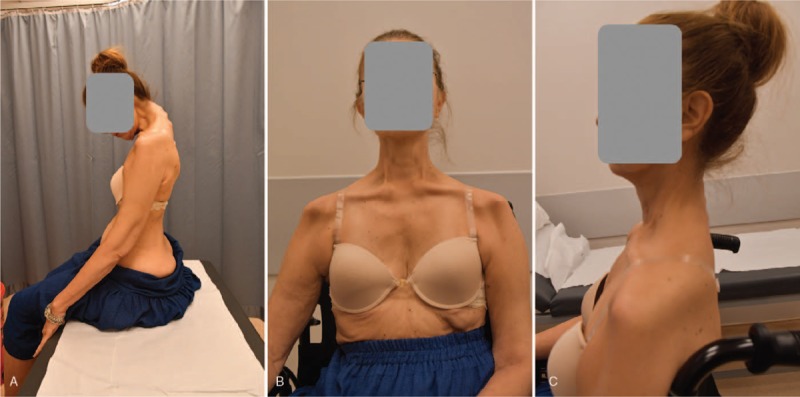
Postoperative X-rays (A and B) and computerized tomography scan (C) showed successful union.

The patient was prepped and draped in prone position under general anesthesia. T2–S1 posterior midline incision was used. Severe paraspinal muscle atrophy was noted (Fig. 2C). Facet joints of all levels between T3 and L5 were bilaterally exposed and joints were removed. Polyaxial pedicle screws (Medtronic, Minneapolis) were introduced to levels from T2 to S1 (17 bilateral levels). Cement was used in T4–5–6, L3–4–5 and iliac screws due to low bone quality. Three rods were used for fixation (Fig. 3). Autograft from spinous processes and 120 cm^3^ spongious allograft were used to facilitate fusion. Three units of packed red blood cells and 2 units of fresh frozen plasma were administered during the operation.

### 2.2 Postoperative care

**Figure 4:**
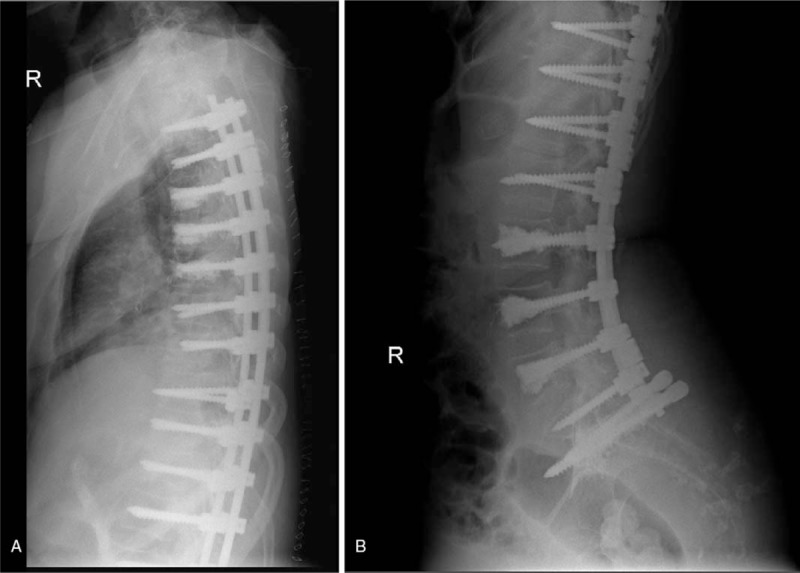
Her postoperative sitting posture was improved dramatically. She was able to sit straight without support (A). To perform an active neck extension, she required a back support (B and C).

The patient was extubated 3 hours after surgery in the intensive care unit. Two units of packed red blood cells and 1 unit of fresh frozen plasma were administered postoperatively on the day of the surgery. Noninvasive respiratory support was utilized following extubation on the day of surgery. Sitting with support was permitted on the day after surgery and without support 1 week after surgery. No problem was observed with wound healing. A soft neck collar was used to support neck extension. The patient was discharged on the 16th day postoperatively. Follow-ups were performed at 3 and 6 months and 1, 2, and 3 years postoperatively. Spinal fusion was confirmed on all levels with CT in the 3rd year. She returned to her professional daily routine 1 month after surgery and preoperative complaints regarding sitting balance, gastrointestinal discomfort, and radicular symptoms recovered completely (Fig. 4).

Postoperative INQoL questionnaire was administered on 3rd year. Postoperative INQoL scores in the symptoms domain were calculated as: I – weakness 94.74 points, II – pain 0 points, III – fatigue 52.63 points, IV – muscle locking 0 points, V – droopy eyelids 0 points, VI – double vision 0 points, VII – swallowing difficulties 0 points. Life domain scores were calculated as: I – activities 57.41 points, II – independence 100 points, III – social relationships 0 points, IV – emotions 0 points, V – body image 61.11 points. Her perceived treatment score was 100 and her quality of life score was 40.

## 3 Discussion

The presented case is the second report in the literature on the spinal fusion of a patient with facioscapulohumeral dystrophy. The patient was diagnosed clinically and was on follow-up since her childhood. Fusion was indicated long after she had become wheelchair bound, which was associated with abdominal discomfort and lower extremity numbness following prolonged sitting. The perioperative period was uneventful and successful fusion was achieved in the first year. Her quality of life was markedly improved at the end of the 3rd year after surgery.

Although spinal deformities in FSHD were mentioned in many studies, surgical treatment has not been studied previously. Lee et al published a case report on pelvic obliquity together with results of gait analysis. The authors discussed spinal and pelvic deformities of this disorder and did not recommend corrective surgeries to avoid any negative effects on walking.^[9]^ Pelvic extensor muscle weakness together with abdominal and core musculature weakness is the main cause of this deformity. Correction may lead to loss of compensation and the ability to walk. Up to 37% of these patients become wheelchair dependent in the course of the disease.^[8]^ Our patient was wheelchair dependent and unable to walk or stand. Surgery was indicated to correct her sitting posture and accompanying sitting discomfort. She was working as a journalist who had to write for several hours every day. Patient reported that her condition affected her daily routine progressively in the last 7 years, with GI discomfort and radicular symptoms with prolonged sitting. She was assessed by internists and no specific GI disorders were found prior to surgery. Her preoperative EMG did not reveal any radicular findings. We had not performed an EMG during and after prolonged sitting. These complaints were resolved immediately after surgery, as spinal instrumentation successfully prevented the deformity. She was able to return to work 1 month after surgery.

Disorders such as Duchenne muscular dystrophy or spinal muscular atrophy are well known conditions causing spinal deformities, especially scoliosis and loss of sitting balance.^[11–14]^ Surgical indications include loss of sitting balance, the magnitude of deformity and progression.^[17]^ It was shown that pulmonary functions were also affected by this condition and improved with surgery.^[17,18]^ To our knowledge, there are no reports on spinal corrective surgery on patients with gastrointestinal disorders or patients with neuromuscular spinal deformity with radicular neuropathy. This case can also be considered as unique with patient complaints.

INQoL questionnaire consists of symptoms, life, treatment, and quality of life subdomains.^[16]^ Symptoms domain has 7 subscales: weakness, pain, fatigue, muscle locking, droopy eyelids, double vision, swallowing difficulties. Life domain has 5 subscales: activities, independence, social relationships, emotions, and body image. Every subscale score except treatment score is calculated on a scale of 0 to 100 with 0 meaning the disease has no impact on her life and 100 meaning the disease makes the most negative impact. For the treatment score, a higher score means a positive impact. We have observed that her pain score under the symptoms domain improved dramatically (94.74–0). As she had to deal with uncomfortable posture while working, level of fatigue after surgery was also improved (94.74–52.63). Her sitting posture was associated with swallowing difficulties, which also recovered completely (63.16–0). FSHD is associated with problems with eye-closure and dryness. Droopy eyelids are not expected in these patients. We believe reported disability was associated with her bent writing posture and computer screen level. Although eyelids were not affected directly from the surgery, increased head level and improved writing comfort likely improved eyelid performance, possible associated with fatigue. Under INQoL's life domain, she reported improvement in activities (76.85–57.41), social relationships (44.44–0), emotions (19.44–0), and body image (88.88–61.11). And finally, the patient reported that her treatment result exceeded her expectations (83.33–100). Overall improvement on the quality of life (55.55–40), as well as improvement in all subdomains showed that spinal fusion was successful in this patient.

Thoraco-lumbo-pelvic spinal fusion can be safely indicated in wheelchair bound patients with facioscapulohumeral dystrophy. This indication should only be reserved for patients without the ability to walk or stand. Hyperlordosis is compensation for pelvic extensor and paraspinal weakness and spinopelvic fusion eliminates this mechanism. Therefore, the results and consequences of this surgery should be discussed in mobile patients.

## Author contributions

**Conceptualization:** Ilker Eren, Mehmet Demirhan.

**Data curation:** Ilker Eren, Berk Abay, Özgür Öztop Çakmak.

**Formal analysis:** Caner Günerbüyük, Özgür Öztop Çakmak.

**Methodology:** Cüneyt Şar.

**Project administration:** Mehmet Demirhan.

**Resources:** Berk Abay.

**Supervision:** Caner Günerbüyük, Cüneyt Şar, Mehmet Demirhan.

**Writing–original draft:** Ilker Eren, Berk Abay, Caner Günerbüyük.

**Writing–review & editing:** Ilker Eren, Özgür Öztop Çakmak, Cüneyt Şar, Mehmet Demirhan.

Ilker Eren: 0000-0003-2965-7690.
